# Relationship between maximum occlusal force and gastrointestinal cancer in community-dwelling older Japanese adults

**DOI:** 10.1038/s41598-021-04158-y

**Published:** 2022-01-10

**Authors:** Takamasa Komiyama, Takashi Ohi, Wakana Ito, Yoshitada Miyoshi, Takako Hiratsuka, Sanae Matsuyama, Ichiro Tsuji, Makoto Watanabe, Yoshinori Hattori

**Affiliations:** 1 Division of Aging and Geriatric Dentistry, Department of Rehabilitation Dentistry, Tohoku University Graduate School of Dentistry, Sendai, Miyagi Japan; 2 Japanese Red Cross Ishinomaki Hospital, Ishinomaki, Miyagi Japan; 3 Division of Epidemiology, Department of Health Informatics and Public Health, Tohoku University School of Public Health, Graduate School of Medicine, Sendai, Miyagi Japan; 4 Institute of Living and Environmental Sciences, Miyagi Gakuin Women’s University, Sendai, Miyagi Japan

**Keywords:** Cancer, Oral diseases, Epidemiology

## Abstract

Globally, the cancer burden is expected to increase as populations are ageing. Therefore, cancer prevention among older age groups is important. This prospective cohort study examined the relationship between the number of remaining teeth, maximum occlusal force, and incidence of gastrointestinal cancer in community-dwelling older Japanese individuals using data from the Tsurugaya project; 847 participants were included. The exposure variables were the number of remaining teeth and the maximum occlusal force, with the outcome being the incidence of gastrointestinal cancer. Covariates were age, sex, medical history, smoking, alcohol consumption, educational attainment, and physical function. The Cox proportional hazard model was used to examine the relationship between the number of remaining teeth, maximum occlusal force, and incidence of gastrointestinal cancer. With a median follow-up of 7.6 years, 63 participants were confirmed to have gastrointestinal cancer. The risk of gastrointestinal cancer was significantly higher in those with an occlusal force lower than the median (hazard ratio, 2.80; 95% confidence interval, 1.54–5.10). No significant risk difference was found according to the number of remaining teeth. Low maximum occlusal force was associated with the incidence of gastrointestinal cancer in community-dwelling older Japanese adults.

## Introduction

The world’s cancer burden is projected to reach 28.4 million cases by 2040, a 47% increase from that in 2020^1^. As populations age worldwide, preventing cancer in older age is one strategy to decrease the global cancer burden, allowing cancer-related medical resources to be allocated toward young and middle-aged populations^2^.

Gastrointestinal cancer, noted for its incidence and death rate in later life, has been associated with oral health in several reports^3,4^. Recently, a prospective nationwide cohort study conducted in South Korea suggested that tooth brushing frequency was associated with the development of gastrointestinal cancer^5^. Another prospective cohort study using data from the United Kingdom Biobank found that self-reported poor oral health (i.e., mouth ulcers, painful gums, bleeding gums, loose teeth, and toothaches) was associated with the incidence of hepatobiliary cancer^6^. Further, several other reports have shown that oral health indicators, such as tooth loss, periodontal disease, and poor oral hygiene, are associated with esophageal, stomach, colorectal, liver, and pancreatic cancers^7–17^. However, these relationships remain controversial because several studies reported contradictory results^18–20^. Previous studies have identified nutritional intake or diet as a risk or suppressive factor for gastrointestinal cancer^21–23^. Oral health indicators, such as the number of remaining teeth and maximum occlusal force, are also associated with nutritional intake or diet, suggesting that these indicators may contribute to an individual’s nutritional wellbeing^24,25^. Therefore, it is rational to hypothesize that these oral health indicators (i.e., the number of remaining teeth and maximum occlusal force) are associated with the incidence of gastrointestinal cancer.

This prospective cohort study investigated the relationship between the number of remaining teeth, maximum occlusal force, and risk of gastrointestinal cancer in community-dwelling older Japanese adults.

## Results

**Figure 1:**
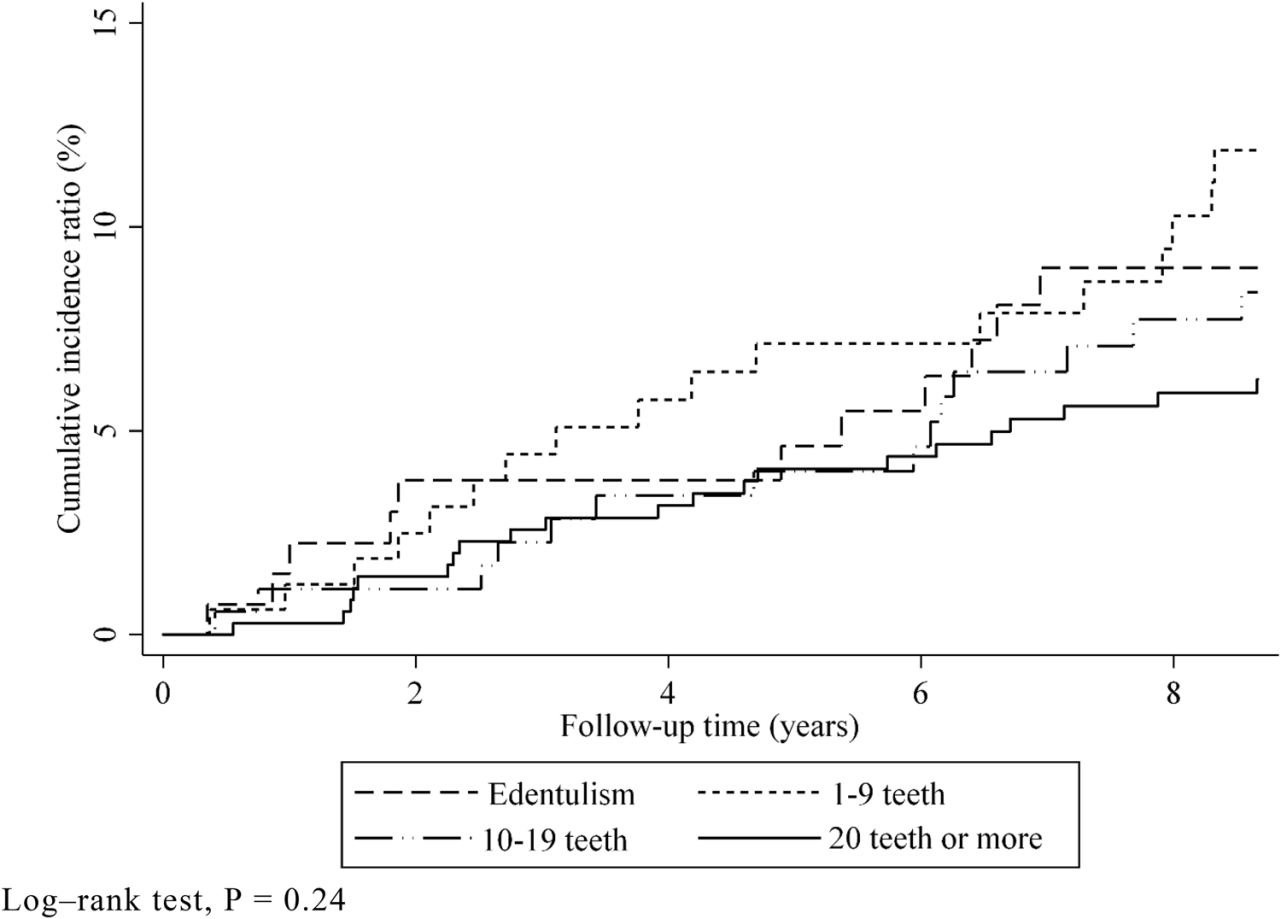
Kaplan–Meier survival curves for the cumulative incidence of gastrointestinal cancer based on the number of remaining teeth (log-rank test, *P* = 0.24).

**Figure 2:**
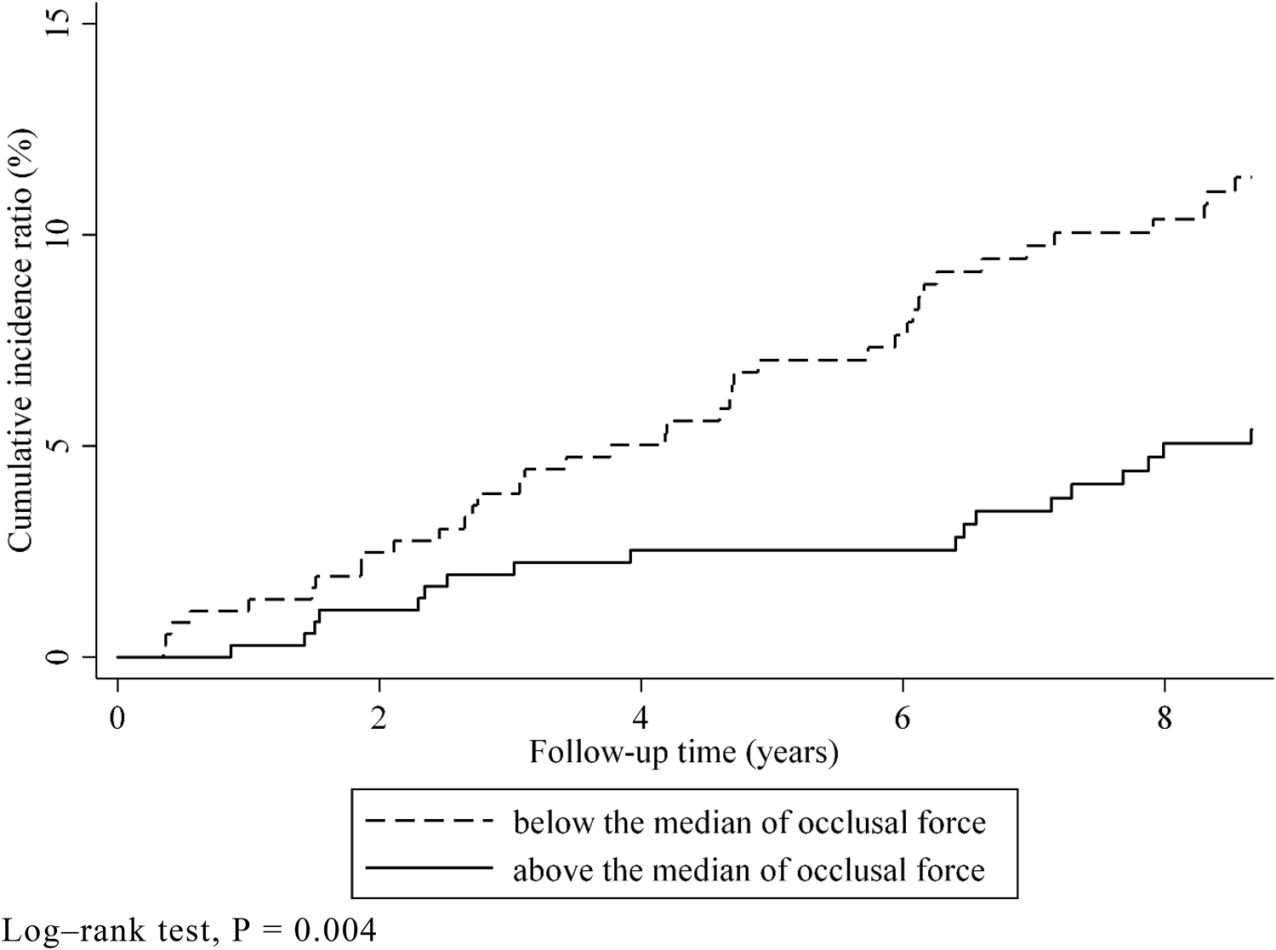
Kaplan–Meier survival curves for the cumulative incidence of gastrointestinal cancer based on the maximum occlusal force (log-rank test, *P* = 0.004).

In total, 847 participants were included in the study. The follow-up comprised 6428 person-years (average, 7.6 years; maximum, 8.6 years), and 63 participants were confirmed to have gastrointestinal cancer. The most frequent gastrointestinal cancer sites were the stomach (n = 24), followed by the colon (n = 11), liver (n = 6), and rectum (n = 6) (Supplementary Table 1). In total, 99 participants died during the follow-up period, and 61 participants had cancer at other sites. Table 1 presents the baseline characteristics based on gastrointestinal cancer. The incidence rate significantly differed based on sex (*P* < 0.001), presence of dyslipidemia (*P* = 0.028), smoking habit (*P* = 0.001), and the maximum occlusal force (*P* = 0.01). The patients included in the analysis and those excluded because of missing data on maximum occlusal force differed in baseline characteristics, such as age (*P* < 0.01), sex (*P* < 0.01), educational attainment (*P* < 0.01), physical function (*P* < 0.01), and alcohol consumption (*P* < 0.01). Figures 1 and 2 present the associations between the cumulative gastrointestinal cancer incidence rate and number of remaining teeth or occlusal force using the Kaplan–Meier method. The cumulative incidence rate of gastrointestinal cancer did not significantly differ among the groups according to the number of remaining teeth (log-rank test, *P* = 0.24) (Fig. 1). The cumulative gastrointestinal cancer incidence ratio significantly differed based on the median occlusal force (above the median, incidence ratio: 5.4%; below the median, incidence ratio: 11.4%; log-rank test, *P* = 0.004) (Fig. 2).

**Table 1:** Baseline characteristics based on the incidence of gastrointestinal cancer.

Variables	Gastrointestinal Cancer				
	Overall	Incidence	No incidence	*P*-value	Incidence rate/1000 person-years
	(N = 847)	(n = 63)	(n = 784)		
Age (in years), mean ± SD	75.4 ± 4.6	76.5 ± 5.3	75.3 ± 4.5	0.125	
Sex, n (%)				< 0.001	
Male	381 (45.0)	44	337		16.1
Female	466 (55.0)	19	447		5.14
Stroke, n (%)	30 (3.5)	2	28	1.000	9.71
Diabetes, n (%)	124 (14.6)	10	114	0.714	10.7
Hypertension, n (%)	348 (41.1)	28	320	0.596	10.6
Dyslipidemia, n (%)	239 (28.2)	10	229	0.028	5.30
Smoking, n (%)				0.001	
Never	488 (57.6)	23	465		5.97
Former	255 (30.1)	32	223		17.3
Current	87 (10.3)	8	79		13.4
Drinking, n (%)				0.056	
Never	341 (40.2)	20	321		7.57
Former	92 (10.9)	11	81		16.3
Current	338 (39.9)	30	308		11.9
< 18 years of educational attainment, n (%)	291 (34.4)	15	276	0.068	6.73
Reduced physical activity*, n (%)	228 (26.9)	12	216	0.353	7.15
Number of remaining teeth, n (%)				0.333	
Edentulism	137 (16.3)	11	126		11.0
1–9	164 (19.5)	17	147		14.3
10–19	181 (21.5)	14	167		9.97
≥ 20	361 (42.8)	21	340		7.47
Maximum occlusal force, n (%)				0.010	
Above the median (≥ 307.2 N)	370 (43.7)	18	352		6.25
Under the median (< 307.2 N)	369 (43.6)	39	330		13.9

Smoking, n = 830; Drinking, n = 771; < 18 years of educational attainment, n = 809; Reduced physical activity, n = 844.

*P*-values were obtained using the Wilcoxon rank-sum test for continuous variables and Fisher’s exact test for categorical variables.

*SD* standard deviation.

*Medical Outcome Study score below 5.

Table 2 outlines the relationship between the oral health indicators and incidence of gastrointestinal cancer. Compared with individuals with ≥ 20 teeth, those with 10–19 teeth, 1–9 teeth, and edentulism did not have a high hazard ratio (HR) in any model. In Model 2, compared with those with ≥ 20 teeth, the HR of individuals with 10–19 teeth was 1.25 (95% confidence interval [CI], 0.62–2.52), that of those with 1–9 teeth was 1.79 (95% CI, 0.90–3.55), and that of those with edentulism was 1.68 (95% CI, 0.77–3.68). Individuals with occlusal force scores below the median had significantly higher HRs in all models than those with scores above the median. The HR of those with an occlusal force score below the median in Model 2 was 2.80 (95% CI, 1.54–5.10) compared with that of those with an occlusal force score above the median. The analysis of the relationship between each covariate and gastrointestinal cancer using the Cox proportional hazard model showed that the HRs increased significantly with age. On the other hand, compared to males, the HRs of females were significantly lower (Supplementary Table 2). First, a sensitivity analysis was used to examine the relationship between the number of remaining teeth, maximum occlusal force, and gastrointestinal cancer by conducting multiple imputations for missing covariates. In the multivariate model, compared with participants with ≥ 20 teeth, the HR of individuals with 10–19 teeth was 1.22 (95% CI, 0.60–2.46), that of those with 1–9 teeth was 1.83 (95% CI, 0.92–3.63), and that of those with edentulism was 1.68 (95% CI, 0.77–3.70). Individuals with a maximum occlusal force below the median score had significantly higher HRs in Model 3 (below median score: HR, 2.81; 95% CI, 1.54–5.13; Supplementary Table 3) than those with a maximum occlusal force above the median score. In the second sensitivity analysis that excluded participants with incident gastrointestinal cancer diagnosed within one year after the baseline survey, the group with a maximum occlusal force score below the median score remained at increased risk (HR 2.53; 95% CI, 1.36–4.69) (Supplementary Table 4). Additionally, in the third sensitivity analysis that accounted for the competing risk of incident cancer in other sites, the group with a maximum occlusal force score below the median score remained at increased risk (HR 2.57; 95% CI, 1.41–4.68) (Supplementary Table 5).

**Table 2:** Relationships between oral health and the incidence of gastrointestinal cancer.

Oral health variables	Participants, n	Incidence, n	Model 1		Model 2
			HR (95% CI)		
**Number of remaining teeth**					
≥ 20	361	21	Reference		Reference
10–19	181	14	1.21 (0.61–2.42)		1.25 (0.62–2.52)
1–9	164	17	1.90 (0.97–3.70)		1.79 (0.90–3.55)
Edentulism	137	11	1.61 (0.76–3.43)		1.68 (0.77–3.68)
**Maximum occlusal force**					
Above the median (≥ 307.2 N)	370	18	Reference		Reference
Below the median (< 307.2 N)	369	39	2.73 (1.53–4.89) **		2.80 (1.54–5.10) **

Model 1: adjusted for age and sex. Model 2: adjusted for age, sex, medical history (stroke, diabetes, hypertension, and dyslipidemia), smoking, alcohol drinking, educational level, and physical function.

The analyses of number of remaining teeth and maximum occlusal force were conducted separately.

*HR* hazard ratio, *CI* confidence interval.

*P < 0.05; **P < 0.01.

## Discussion

This study elucidated the relationship between a reduced maximum occlusal force and the incidence of gastrointestinal cancer in community-dwelling older Japanese individuals, after adjusting for age, sex, medical history, smoking, alcohol consumption, educational attainment, and physical function. The number of remaining teeth was not associated with gastrointestinal cancer, and a sensitivity analysis confirmed that the relationship between the maximum occlusal force and incidence of gastrointestinal cancer was robust. To the best of our knowledge, this is the first study to examine the relationship between the maximum occlusal force and incidence of gastrointestinal cancer in community-dwelling older adults.

Several prospective cohort studies from the United Kingdom, the United States, and other Asian countries have demonstrated the association of oral health with gastrointestinal or related cancers^5,6,13,17^. This study supports these results with added perspective, as the relationship between oral health and gastrointestinal cancer was confirmed purely in community-dwelling older adults.

To date, studies exploring the risk factors or predictors of gastrointestinal or related cancers have been applied as oral health indicators; mainly for periodontal disease or oral hygiene^5,6,12,13,16,19^. Tooth loss partially reflects information about past periodontal disease or oral health behavior and has also become a surrogate masticatory performance indicator^26^. In this study, tooth loss was not associated with the incidence of gastrointestinal cancer in the primary analysis and in the sensitivity analysis. We cannot assume that information about the number of remaining teeth indicates periodontal disease, masticatory performance, or oral health behavior in this study. However, we investigated the maximum occlusal force, which is an indirect indicator of masticatory performance^27^, and confirmed that reduced occlusal force was related to the incidence of gastrointestinal cancer. Currently, the biological mechanism linking maximum occlusal force with gastrointestinal cancer remains uncertain; however, nutritional intake or diet is a possibility. For individual cancers, fresh vegetable consumption was associated with a low incidence of gastric cancer^28^, and the intake of dietary fibers was related to a reduced colorectal cancer risk^29^. Further, a vegetable-based diet was associated with a lower risk of liver cancer^22^, and vegetable and fruit intake were associated with reduced risks of esophageal, biliary duct, and pancreatic cancers^30–32^. Regarding the gut microbiome, abundant microbiotas in the intestines compete with each other and are affected by dietary intake. A previous meta-analysis elucidated that long-term vegetable or fruit intake promotes the superiority of anti-inflammatory-related species over pro-inflammatory-related species in the gut. Gut dysbiosis and its related inflammation have also been considered a risk for cancer^33^. Generally, many vegetables and fruits have a hard texture in their raw forms. In fact, previous reports have shown that impaired masticatory performance was associated with a lower intake of dietary fibers, vegetables, and fruits^24,34,35^.

This study has some limitations. First, the sample size was small. Therefore, a subgroup analysis examining the association between oral health status and site-specific cancers was not conducted. Using site-specific cancer incidences as the primary outcome may help elucidate the mechanism between the maximum occlusal force and gastrointestinal cancer. Second, the generalizability of these results is limited because this study targeted Japanese individuals alone and the response rate was not high; therefore, it is unclear whether these results apply to other regions and other older populations. Third, as a relatively high number of participants were excluded from the analysis because of missing information regarding the maximum occlusal force, information bias might have occurred. Fourth, this study evaluated oral health status in a baseline survey. It remains unclear whether long-term deterioration of oral health status is related to the incidence of gastrointestinal cancer. If nutritional intake or diet is mediated in this relationship, one would need to observe the oral health status and diet for a long period. Finally, in the present study, functional teeth could not be considered as an exposure variable because 96.5% of the participants had 20 functional teeth or more. To strengthen the hypothesis that nutritional intake or diet mediates the relationship between masticatory performance and incidence of gastrointestinal cancer, future research is required in which analyses of functional teeth and gastrointestinal cancer would be performed.

In conclusion, this prospective cohort study identified the maximum occlusal force as a risk factor for gastrointestinal cancer in community-dwelling older Japanese adults. As with other oral health indicators, such as periodontal disease and oral hygiene, maximum occlusal force could be an indicator for gastrointestinal cancer risk. Finally, elucidating the underlying biological mechanism is important for understanding the relationship between occlusal force and the incidence of gastrointestinal cancer.

## Methods

The current study followed the Strengthening the Reporting of Observational Studies in Epidemiology (STROBE) Statement guidelines^36^ and was performed in accordance with the Helsinki Declaration.

### Study design

This prospective cohort study was conducted as a part of the Tsurugaya project. The project, conducted in 2003, aimed to perform a comprehensive geriatric assessment of community-dwelling older adults in Tsurugaya district, a suburban area of Sendai City in Japan, with a growing aging population. Eligibility criteria were community-dwelling older adults aged ≥ 70 years in Tsurugaya district in 2003; they were invited by mail to participate in the Tsurugaya project. The baseline survey included the maximum occlusal force and number of remaining teeth as exposure variables, and the primary outcome was gastrointestinal cancer based on medical records, which were collected until February 2012 using the National Health Insurance record.

### Study participants

**Figure 3:**
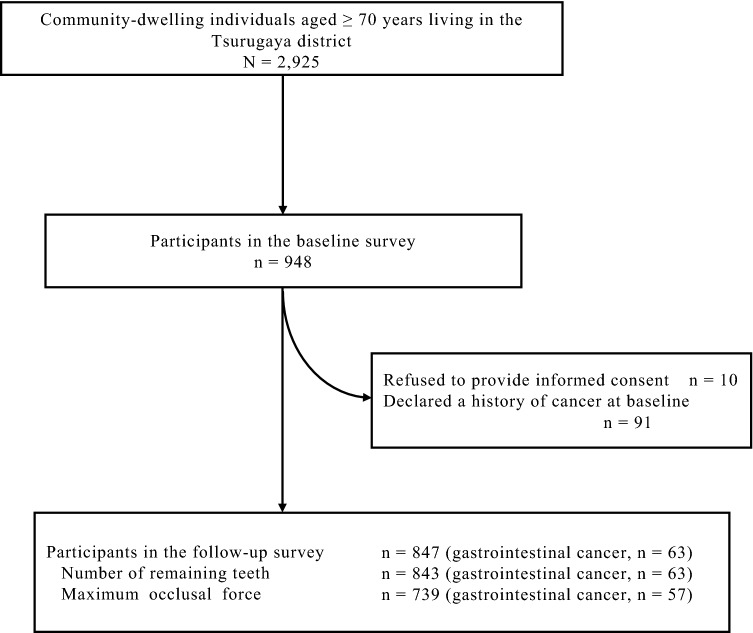
Flow diagram of the study participants.

Previous studies have described the Tsurugaya project^37,38^. Figure 3 presents a flow diagram of the participants. The target population was 2925 individuals aged ≥ 70 years who resided in the Tsurugaya district. Of these, 948 participants received the baseline assessment in July 2003. Ten participants were excluded from the study, as they did not provide written informed consent to participate in this project. Ninety-one participants with a history of cancer at baseline were further excluded. Finally, 847 participants were included in the study, and their cancer data were analyzed. They provided written informed consent regarding baseline data and follow-up data (date and incidence of death and gastrointestinal cancer) during the baseline survey. The study protocol was approved by the Institutional Review Board of the Tohoku University Graduate School of Medicine (2002–040, 2017–1-312).

### Assessment of dental status

Well-trained dentists performed dental examinations and evaluated the number of remaining teeth^39^. The participants were divided into groups based on the number of remaining teeth: edentulism, 1–9 teeth, 10–19 teeth, and ≥ 20 teeth. Residual tooth roots were excluded from the total number of teeth. The maximum bilateral occlusal force is considered an indirect indicator of masticatory performance^27^ and is often used to evaluate oral hypofunction in Japanese clinical settings^40^. The occlusal force was measured using a Dental Prescale I 50H R-type film (Fuji Photo Film Co., Tokyo, Japan) and Occluzer (FDP-705; Fuji Photo Film Co., Tokyo, Japan). The film was set on the participant’s mandibular dentition, after which the participant was instructed to voluntarily and to maximally clench their jaws for 3 s. Participants with removable dentures were asked to keep the dentures in their mouths during the examination. The participants were divided into two groups based on the median maximum occlusal force score (i.e., those with scores above or below the median)^41^.

### Assessments of covariates

The baseline survey also assessed covariates such as medical history (stroke, diabetes, hypertension, and dyslipidemia), smoking and drinking habits, educational attainment, and physical function. Smoking and drinking habits were independently classified as current, former, or never. Educational attainment was considered a socioeconomic position and classified based on the age at graduation from the last school they attended (age < 18 years or ≥ 18 years). Physical function was assessed using the six-item Physical Function Scale of the Short-Form General Health Survey adapted from the Medical Outcome Study (MOS)^42^. Participants were classified into two groups based on the MOS score: those capable of performing vigorous physical activity (scores 5 or 6) and those capable of performing moderate or low physical activity (scores 0–4)^43^.

### Follow-up and outcome

The primary outcome was the incidence of gastrointestinal cancer during the follow-up period, from July 2003 to February 2012. The incidence of gastrointestinal cancer and mortality were investigated in all individuals using the National Health Insurance (NHI) claims history files, which form part of the Japanese medical insurance system, and contain data, including the number of outpatient visits, hospitalizations, and death, from the Miyagi NHI Association. If the NHI claims record confirmed gastrointestinal cancer, the hospital attended by the patient was identified, and a researcher visited the hospital to review the records and to confirm the cancer diagnosis and dates. Gastrointestinal cancer was classified according to the International Classification of Diseases codes from C15 to C26^6^.

### Statistical analyses

The participants’ baseline characteristics for the incidence of gastrointestinal cancer were evaluated using the Wilcoxon rank-sum test for continuous variables and Fisher’s exact test for categorical variables. The relationship between the number of remaining teeth, maximum occlusal force, and gastrointestinal cancer incidence rate was assessed using Kaplan–Meier survival curves and log-rank tests. If the participant died or developed cancer at another site during the follow-up period, the case was censored. Cox proportional hazard models were used to calculate the HRs and 95% CIs. Two models were created; Model 1 included age and sex, and Model 2 included age, sex, medical history (stroke, diabetes, hypertension, and dyslipidemia), smoking history (current, former, or never), alcohol consumption (current, former, or never), educational attainment (< 18 or ≥ 18 years), and physical function (vigorous activity or low activity) to address confounders. In the Cox proportional hazard model, analyses of the number of remaining teeth and maximum occlusal force were performed independently. Missing data were recorded as “missing” categories in categorical variables to maximize the sample size of participants included in the analysis.

Several sensitivity analyses were conducted for the subsidiary. First, there were multiple imputations for missing covariate values. Missing values were categorized as “missing at random” and were given multiple imputations by chained equations. The procedure substituted the missing covariate value with the most likely score and created five output datasets. The pooled HRs and 95% CIs for gastrointestinal cancer were calculated using the multivariate-adjusted Cox model after imputing the missing covariate data. Second, the Cox proportional hazard model was conducted by excluding the participants who were diagnosed with gastrointestinal cancer within one year after the baseline survey. Third, the Fine-Gray model was conducted to account for the competing risk of incident cancer at other sites. All data analyses were conducted using Stata version 15.1 (Stata Corp, College Station, TX, USA). All analyses were performed as two-tailed tests and P-values < 0.05 were considered statistically significant.

## Supplementary Information

Supplementary Information.

## Data Availability

No datasets were generated or analyzed during the current study.
