# Supplementary material for: Relationship between maximum occlusal force and gastrointestinal cancer in community-dwelling older Japanese adults
Source: Sci Rep. 2022 Jan 10;12:440. doi: 10.1038/s41598-021-04158-y (PMC8748517; doi:10.1038/s41598-021-04158-y)
Supplement: Supplementary file 1 — Supplementary Information. [file 41598_2021_4158_MOESM1_ESM.docx]

**Supplementary Information**

**Relationship between maximum occlusal force and gastrointestinal cancer in community-dwelling older Japanese adults**

Takamasa Komiyama^a^*, Takashi Ohi^a, b^, Wakana Ito^a^, Yoshitada Miyoshi^a^, Takako Hiratsuka^a^, Sanae Matsuyama^c^, Ichiro Tsuji^c^, Makoto Watanabe^d^, Yoshinori Hattori^a^

**Supplemental Table 1.** Gastrointestinal cancer sites according to ICD-10

|  | n = 63 |
| --- | --- |
| Gastrointestinal Cancer Site |  |
| Stomach (C16) | 24 |
| Colon (C18, C19) | 11 |
| Liver (C22) | 6 |
| Rectum (C20) | 6 |
| Biliary tract (C23, C24) | 5 |
| Small intestine (C17) | 4 |
| Esophageal (C15) | 3 |
| Pancreatic (C25) | 3 |
| Other and ill-defined digestive organs (C26) | 1 |

ICD-10, 10^th^ version of the International Classification of Diseases

**Supplemental Table 2.** Relationship covariates and incidence of gastrointestinal cancer

|  | Model |
| --- | --- |
| variables | HR (95% CI) |
| Age (with an increase in age of one year) | 1.07 (1.02–1.12) ^*^ |
| Sex |  |
| Male | Reference |
| Female | 0.30 (0.18–0.50) ^**^ |
| Stroke  Yes | Reference |
| No | 0.81(0.29–2.22) |
| Diabetes |  |
| Yes | Reference |
| No | 1.15 (0.59–2.27) |
| Hypertension |  |
| Yes | Reference |
| No | 0.94 (0.60–1.49) |
| Dyslipidemia |  |
| Yes | Reference |
| No | 1.56 (0.85–2.86) |
| Smoking |  |
| Never | Reference |
| Former | 1.34 (0.69–2.60) |
| Current | 1.24 (0.52–2.93) |
| Drinking |  |
| Never | Reference |
| Former | 1.16 (0.55–2.44) |
| Current | 0.96 (0.53–1.72) |
| Educational attainment |  |
| ≥ 18 years | Reference |
| < 18 years | 1.46 (0.85–2.49) |
| Physical activity |  |
| Vigorous | Reference |
| Reduced | 1.36 (0.74–2.48) |

CI, confidence interval; HR, hazard ratio

*P < 0.05,　**P < 0.01.

Each variable was adjusted for age and sex. Age was adjusted for sex; sex was adjusted for age.

**Supplemental Table 3.** Relationship between oral health and incidence of gastrointestinal cancer after multiple imputation

|  |  |  | Model 1 | Model 2 | Model 3 |
| --- | --- | --- | --- | --- | --- |
| Oral health variables | Participants, n | Incidence, n | HR (95% CI) | | |
| Number of remaining teeth |  |  |  |  |  |
| ≥ 20 | 361 | 21 | Reference | Reference | Reference |
| 10–19 | 181 | 14 | 1.33  (0.68–2.63) | 1.21  (0.61–2.42) | 1.22  (0.60–2.46) |
| 1–9 | 164 | 17 | 1.92  (1.01–3.64) ^*^ | 1.90  (0.97–3.70) | 1.83  (0.92–3.63) |
| Edentulism | 137 | 11 | 1.48  (0.72–3.08) | 1.61  (0.76–3.43) | 1.68  (0.77–3.70) |
| Maximum occlusal force |  |  |  |  |  |
| Above the median  (≥ 307.2N) | 370 | 18 | Reference | Reference | Reference |
| Below the median (< 307.2N) | 369 | 39 | 2.23  (1.27–3.89) ^**^ | 2.73  (1.53–4.89) ^**^ | 2.81  (1.54–5.13) ^**^ |

CI, confidence interval; HR, hazard ratio

*P < 0.05,　**P < 0.01.

Model 1: null model. Model 2: adjusted for age and sex. Model 3: adjusted for age, sex, medical history (stroke, diabetes, hypertension, and dyslipidemia), smoking, alcohol drinking, educational level, and physical function.

**Supplemental Table 4.** Relationship between oral health and incidence of gastrointestinal cancer excluding individuals with incident gastrointestinal cancer within one year

|  |  |  | Model 1 | Model 2 | Model 3 |
| --- | --- | --- | --- | --- | --- |
| Oral health variables | Participants, n | Incidence, n | HR (95% CI) | | |
| Number of remaining teeth |  |  |  |  |  |
| ≥ 20 | 360 | 20 | Reference | Reference | Reference |
| 10–19 | 179 | 13 | 1.31  (0.65–2.63) | 1.18  (0.58–2.42) | 1.22  (0.59–2.52) |
| 1–9 | 162 | 15 | 1.79  (0.92–3.50) | 1.77  (0.88–3.57) | 1.71  (0.83–3.50) |
| Edentulism | 135 | 9 | 1.28  (0.58–2.82) | 1.40  (0.62–3.16) | 1.45  (0.62–3.35) |
| Maximum occlusal force |  |  |  |  |  |
| Above the median  (≥ 307.9N) | 368 | 17 | Reference | Reference | Reference |
| Below the median (< 307.9N) | 367 | 35 | 2.09  (1.17–3.74) ^*^ | 2.53  (1.39–4.62) ^**^ | 2.53  (1.36–4.69) ^**^ |

CI, confidence interval; HR, hazard ratio

*P < 0.05,　**P < 0.01.

Model 1: null model. Model 2: adjusted for age and sex. Model 3: adjusted for age, sex, medical history (stroke, diabetes, hypertension, and dyslipidemia), smoking, alcohol drinking, educational level, and physical function.

**Supplemental Table 5.** Relationship between oral health and incidence of gastrointestinal cancer accounting for the incidence cancer at other sites as competing risk

|  |  |  | Model 1 | Model 2 | Model 3 |
| --- | --- | --- | --- | --- | --- |
| Oral health variables | Participants, n | Incidence, n | HR (95% CI) | | |
| Number of remaining teeth |  |  |  |  |  |
| ≥ 20 | 361 | 21 | Reference | Reference | Reference |
| 10–19 | 181 | 14 | 1.36  (0.69–2.67) | 1.24  (0.60–2.56) | 1.29  (0.62–2.69) |
| 1–9 | 164 | 17 | 1.89  (1.00–3.58) | 1.87  (0.96–3.63) | 1.76  (0.93–3.36) |
| Edentulism | 137 | 11 | 1.49  (0.72–3.10) | 1.55  (0.72–3.32) | 1.66  (0.77–3.60) |
| Maximum occlusal force |  |  |  |  |  |
| Above the median  (≥ 307.2N) | 370 | 18 | Reference | Reference | Reference |
| Below the median (< 307.2N) | 369 | 39 | 2.14  (1.20–3.82) ^*^ | 2.57  (1.40–4.72) ^**^ | 2.57  (1.41–4.68) ^**^ |

CI, confidence interval; HR, hazard ratio

*P < 0.05,　**P < 0.01.

Model 1: null model. Model 2: adjusted for age and sex. Model 3: adjusted for age, sex, medical history (stroke, diabetes, hypertension, and dyslipidemia), smoking, alcohol drinking, educational level, and physical function.
